# Supplementary material for: A Likelihood-Based Framework for Simultaneously Learning Both Noise and Growth Dynamics Using Biologically-Informed Neural Networks
Source: Bull Math Biol. 2026 Oct 11;88(11):201. doi: 10.1007/s11538-026-01755-8 (PMC13654870; doi:10.1007/s11538-026-01755-8)
Supplement: Supplementary file 1 [file 11538_2026_1755_MOESM1_ESM.pdf]

Supplementary Figures

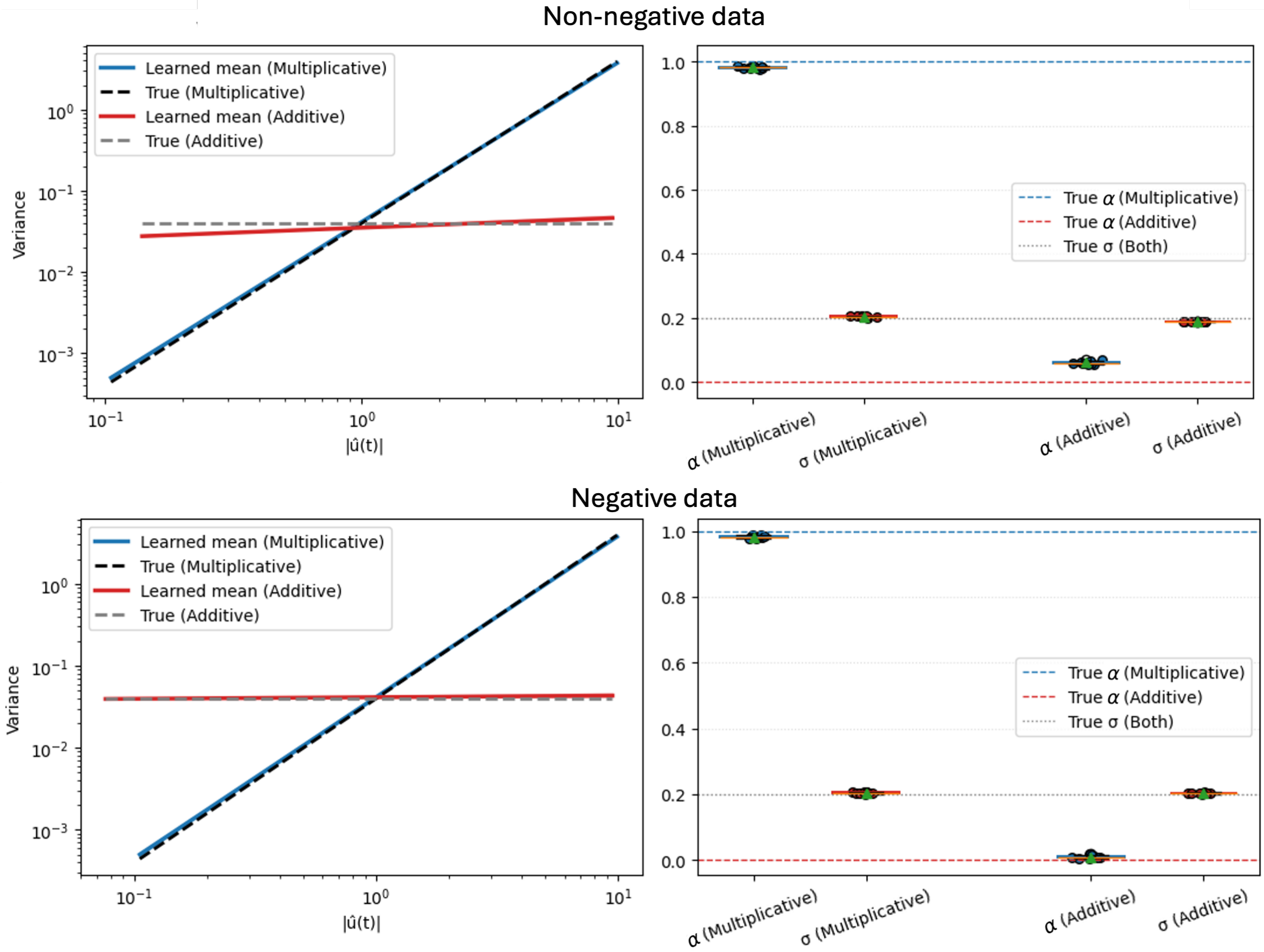

Figure S1: Left: learned variance $\sigma^2(u)$ as a function of the population density, u, with true underlying constant variance for additive noise ($\alpha = 0$, red) and state-dependent scaling for multiplicative noise ($\alpha = 1$, blue). Right: inferred noise parameters (σ_0, α) across ten ensemble runs plotted against their true values (additive in red, multiplicative in blue). The top row shows example results for one hundred and one time points of input data that did not contain any negative values. The bottom row shows the same results when negative densities were allowed. These results demonstrate that the discrepancies observed in the results for additive data arise as a result of the data structure, rather than the BINN framework itself.

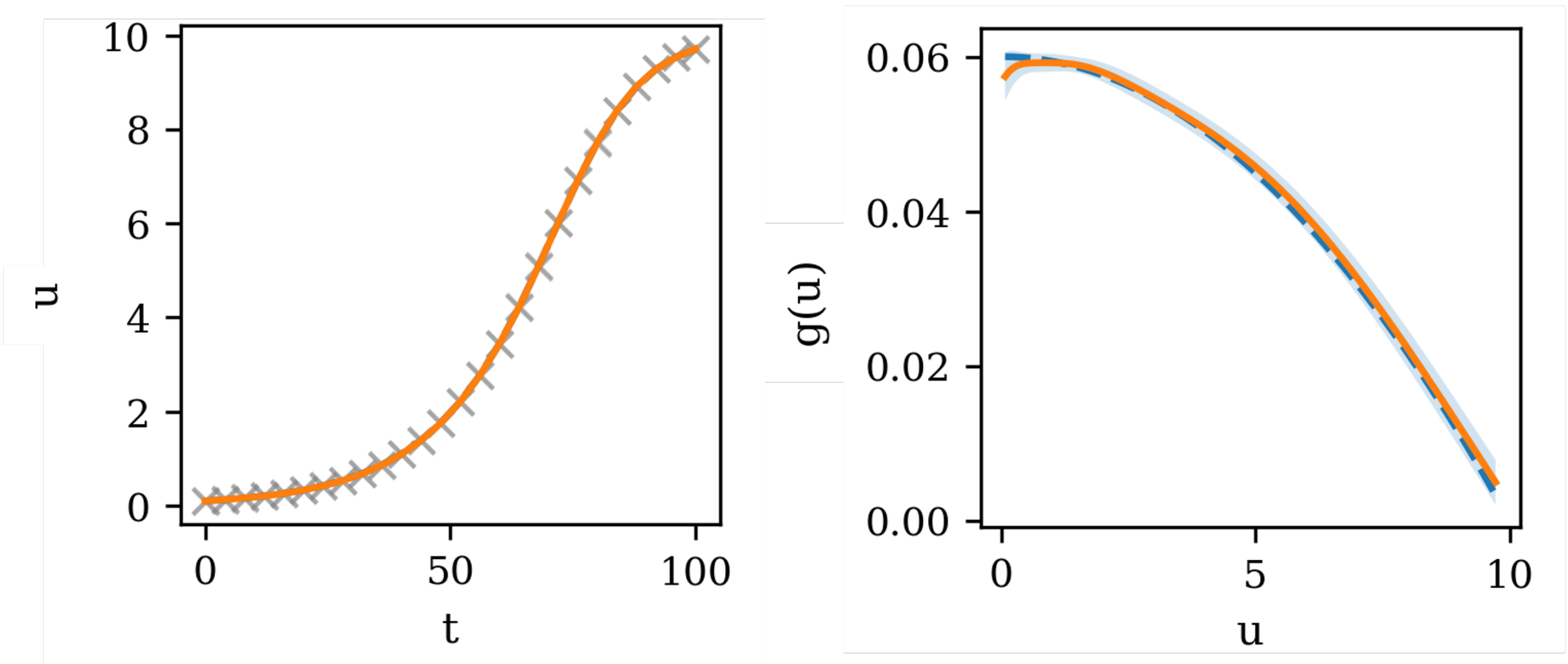

Figure S2: Plots demonstrating the recovery of the population dynamics and growth law using the NLL–BINN framework for synthetic data generated from the Richards' model (Section 3) with parameters r, K, u_0, and ν as specified in Section 3.1. Noise was added according to Equation (3), using $\alpha = 0$ and $\sigma_0 = 0.01$. Left: mean learned trajectory $u_\theta(t)$ in solid orange lines, with one standard deviation (light blue) and noisy observations from all ten replicates (grey). Right: inferred crowding functions $g_\phi(u)$ with the mean shown in orange, the truth as a dashed blue line and the variability shown by blue shading.
